# PIM2 promotes hepatocellular carcinoma tumorigenesis and progression through activating NF-κB signaling pathway

**DOI:** 10.1038/s41419-020-2700-0

**Published:** 2020-07-02

**Authors:** Xuming Tang, Tingting Cao, Yun Zhu, Liyi Zhang, Jinna Chen, Tengfei Liu, Xiaoyan Ming, Shuo Fang, Yun-fei Yuan, Lingxi Jiang, Jian-Dong Huang, Xin-Yuan Guan

**Affiliations:** 1 School of Biomedical Science, Li Ka Shing Faculty of Medicine, The University of Hong Kong, Hong Kong, China; 2 Department of Clinical Oncology, Li Ka Shing Faculty of Medicine, The University of Hong Kong, Hong Kong, China; 3 Weil Medical College of Cornell University New York, New York, NY USA; 4 State Key Laboratory for Liver Research, Li Ka Shing Faculty of Medicine, The University of Hong Kong, Hong Kong, China; 5 State Key Laboratory of Oncology in Southern China, Sun Yat-Sen University Cancer Center, Guangzhou, China; 6 Department of General Surgery, Ruijin Hospital, Shanghai Jiao Tong University School of Medicine, Shanghai, China

**Keywords:** Liver cancer, Apoptosis, Cell invasion

## Abstract

Inflammatory factors and activation of oncogenes both played critical roles in the development and progression of human hepatocellular carcinoma (HCC). However, the interplay between these two has not been well studied. In this study, we found that regulated by TNFα, Pim-2 proto-oncogene, serine/threonine kinase (PIM2) was highly expressed in HCC and correlated with poor prognosis (*P* = 0.007) as well as tumor recurrence (*P* = 0.014). Functional studies showed that PIM2 could enhance abilities of cell proliferation, cell motility, angiogenesis, chemo-resistance, and in vivo tumorigenicity and HCC metastasis. Mechanistic studies revealed that PIM2 could activate NF-κB signaling pathway through upregulating phosphorylation level of RIPK2. Interestingly, TNFα treatment could induce the expression of PIM2, and overexpression of PIM2 could in turn upregulate the expression of TNFα in HCC cells. More importantly, we found the expression level of PIM2 increased with the progression of liver cirrhosis, and PIM kinase inhibitor AZD1208 treatment could effectively attenuate HCC cells’ tumorigenic ability both in vitro and in vivo. Collectively, our study revealed the interaction between an inflammatory factor and a proto-oncogene that contributed to tumorigenesis and progression of HCC, and PIM kinase inhibition may serve as a therapeutic target in the treatment of HCC.

## Introduction

Hepatocellular carcinoma (HCC) is one of the most common human malignancies as well as one of the leading causes of cancer related mortality worldwide^1^. Major risk factors for HCC development, include chronic infection of hepatitis B/C viruses, alcohol consumption, and aflatoxin intake^2^. The close association between HCC and chronic hepatitis is well established according to etiological studies, and approximately 80% HCC patients have hepatitis history^3^. Many genetic and epigenetic changes have been associated with the development and progression of HCC, such as activation of oncogenes *CHD1L*^4^ and *SPOCK1*^5^, and inactivation of tumor suppressor genes *TAT*^6^ and *OSGIN1*^7^.

Previously, RNA sequencing (RNA-Seq) was applied to identify genetic alterations between three pairs of HCC tumor and corresponding nontumor samples^8^, and overexpression of PIM kinase family members (including *PIM1*–*PIM3*) were observed in HCC tumor tissues, among which *PIM2* was the most significantly upregulated one. It has been demonstrated that TNFα and IL-6 played pivotal roles in inflammation induced HCC in genetic modified and dietary obesity mouse models^9^. Further studies found that the expression of *PIM2* could be regulated by various growth factors and cytokines, including TNFα and IL-6^10–12^. Therefore, we hypothesize that PIM2 may play an important role in inflammation induced hepatocarcinogenesis.

*PIM2* proto-oncogene belongs to a serine/therine kinase family that was firstly identified as proviral insertion site in moloney murine leukemia virus infection-induced lymphoma in mouse models in the 1980s^13^. In the present study, *PIM2* was found frequently upregulated in HCCs and *PIM2* upregulation was significantly associated with HCC recurrence and poorer prognosis. Both in vitro and in vivo functional assays demonstrated the oncogenic ability of PIM2, and the underlying molecular mechanism was also revealed.

## Material and methods

### HCC clinical samples and cell lines

A total of 134 paired HCC specimens (tumor and paired adjacent nontumor tissues) were obtained from patients who underwent hepatectomy from HCC at Sun Yat-Sen University Cancer Center (Guangzhou, China). Two immortalized hepatocyte cell lines and HCC cell lines used in this study have been described previously^8,14^. All cell lines were tested for the absence of mycoplasma contamination and authenticated by morphologic observation (MycoAlert, Lonza, Switzerland) 3 months ago. See the Supplementary Materials and Methods section for details.

### Plasmid constructs and lenti-virus transduction

Full-length of human *PIM2* gene was PCR amplified and cloned into pLenti6/v5-D-topo expression vector (Invitrogen) according to manufacturer’s instructions. *PIM2* containing lenti-virus was then stably transduced into HCC cell lines by blasticidin selection. Empty vector transduced cells were used as controls. Two short hairpin RNAs (shRNA) specifically targeting on *PIM2* or specifically targeting on *RIPK2* were cloned into pLL3.7 lenti-viral vector. HCC cell lines were transduced with shRNAs to establish stable knockdown cell lines. See the Supplementary Materials and Methods section for details.

### Flow cytometry

Cells were treated with 5-FU or cisplatin for 48 h and were collected for flow cytometry analysis after staining with Annexin-V–fluorescein isothiocyanate and propidium iodide (PI) using the Annexin-V–Fluos Staining Kit (Roche).

### Immunofluorescence (IF) staining and confocal microscopy

Cells were transiently transfected with Flag-tagged PIM2, and 48 h later, cells were fixed, permeabilized, and blocked. Primary antibodies were incubated at 4 °C overnight, then cells were thoroughly washed and followed by incubation with secondary antibodies. The nuclei was stained with DAPI Invitrogen, CA). Images were captured using a confocal laser scanning microscope (Zeiss LSM510 META). See the Supplementary Materials and Methods section for detailed experimental procedures.

### Functional assays

See the Supplementary Materials and Methods section for detailed experimental procedures of in vitro and vivo functional assays.

### RNA extraction and qRT-PCR

Total RNA was extracted using the TRIZOL Reagent (Invitrogen) and reverse transcription was performed. The cDNA was subjected to quantitative real-time PCR (qRT-PCR) using the SYBR Green PCR Kit (Applied Biosystems, Carlsbad, CA). The relative levels of expression were quantified and analyzed. See the Supplementary Materials and Methods section for detailed experimental procedures and the primer sequences.

### Antibodies and western blotting

Western blot analysis was performed according to the standard protocol. Information of the antibodies for Western blot is listed in the Supplemental Materials and Methods.

### Dual-luciferase reporter assay

To evaluate activity of NF-κB signaling pathway, 100 ng of pNFκB-Luc and 20 ng of Renilla luciferase reporter plasmids were transiently co-transfected into cells seeded in 96-well white plates (SPL, Gyeonggi-do, Korea). Forty-eight hour after plasmids transfection, the transfected cells were lysed and luciferase activity was assessed by the Dual-Glo Luciferase Reporter Assay System (Promega Corporation, Madison, WI, USA).

### IHC and H&E staining

IHC and H&E staining were performed as previously described^8^. Information of the antibodies for IHC staining is listed in the Supplemental Materials and Methods.

### Migration and invasion assays

See the Supplementary Materials and Methods section for detailed experimental procedures of in vitro and vivo metastasis assays.

### Drug sensitivity assays

Cells were seeded in 96-well plates at a density of 5 × 10^3^ cells per well. After 48 h treatment using the chemotherapeutic agent cisplatin or 5-FU at different concentrations, cell viability was detected by XTT Cell Proliferation Assay (Roche Diagnostics). The data represent three independent experiments.

### Statistical analysis

See the Supplementary Materials and Methods section for details.

## Results

### PIM2 is frequently upregulated in HCC patients and correlated with poor prognosis

**Fig. 1:**
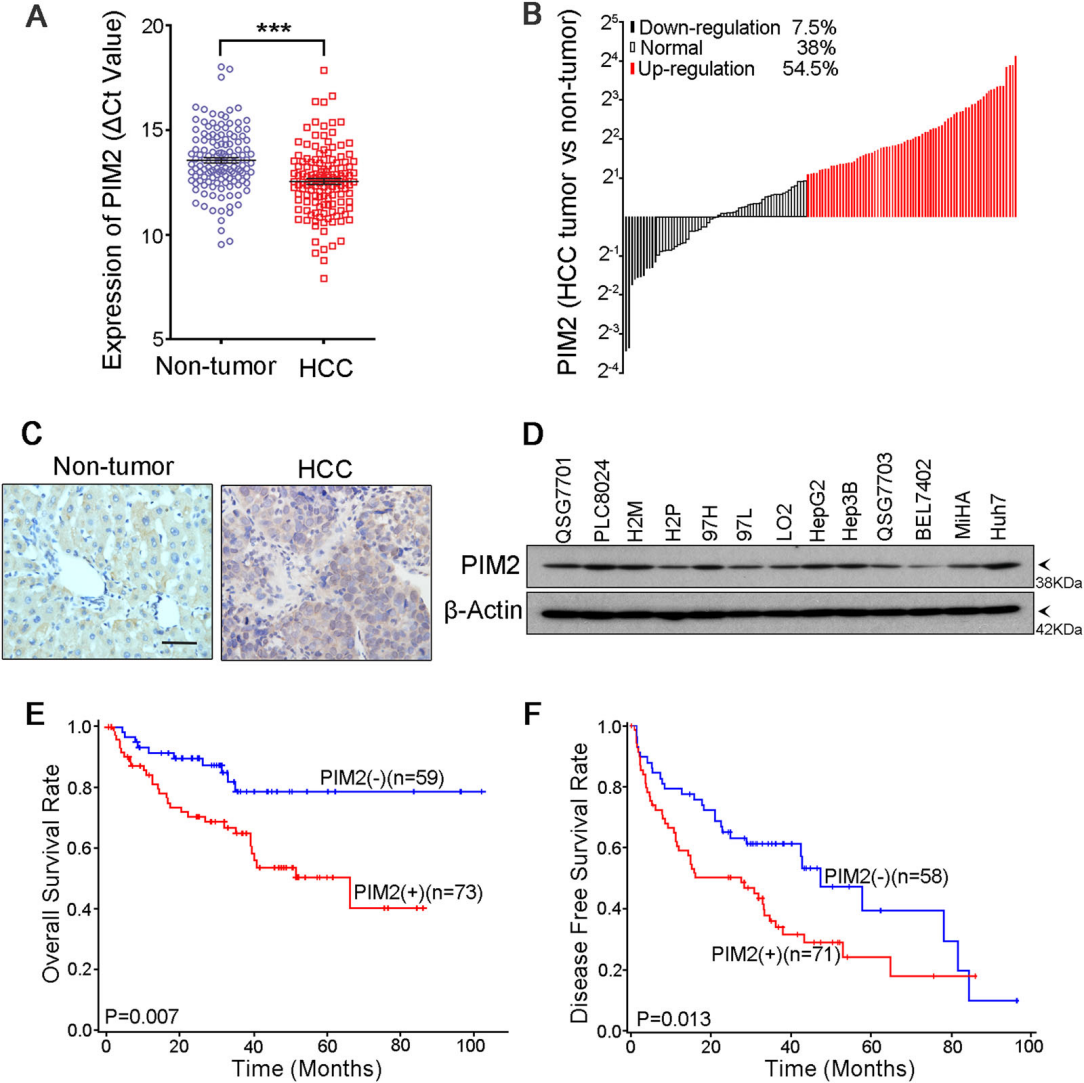
PIM2 is frequently upregulated in HCC patients. **a** Relative expression of PIM2 in 134 pairs of paired HCC tumor and nontumor tissues detected by qRT-PCR. **b** Ratio of PIM2’s relative expression level between paired HCC tumor and nontumor tissue. Upregulation or downregulation are defined as more than twofold change. **c** Representative image of IHC staining of PIM2’s expression in paired HCC tumor and nontumor tissue. Original magnification, ×400. **d** Expression of PIM2 in HCC cell lines detected by Western Blot. **e** Kaplan–Meier overall survival curve and **f** disease-free survival curve of HCC patients correlated with the expression of PIM2. PIM2 (+), patients with PIM2 upregulation; PIM2 (−), patients without PIM2 upregulation.

In the present study, expression of *PIM2* was compared between tumor and corresponding adjacent nontumor tissues by qRT-PCR in 134 primary HCCs. The average ΔCt value of *PIM2* in HCC tumor tissues was significantly lower than that in nontumor tissues (*P* < 0.001, paired Student *t* test, Fig. 1a), indicating that the relative expression level of *PIM2* was dramatically higher in tumor tissues. Upregulation of *PIM2* (defined as >2-fold increase in tumor tissues compared with paired nontumor tissues) was detected in 73/134 (54.5%) of HCCs (Fig. 1b). Upregulation of PIM2 in protein level was detected in 9/16 (56.3%) of HCC cases by western blot analysis (Supplementary Fig. S1A), and confirmed by IHC staining (Fig. 1c). Expression levels of PIM2 in two immortalized hepatocyte cell lines (MiHA and LO2) and 11 HCC cell lines were also detected by western blot analysis (Fig. 1d).

### Clinical significance of PIM2 upregulation in HCC patients

To analyze the association of *PIM2* upregulation with clinicopathological features in 134 HCC patients, HCC patients were divided into two groups with twofold increase as the cut-off line. The results showed that upregulation of *PIM2* was significantly correlated with vascular invasion (*P* = 0.025), recurrence (*P* = 0.014), and TNM stage (*P* = 0.013) (Supplemental Table 1). More importantly, upregulation of *PIM2* was significantly associated with shorter overall survival time (*P* = 0.007) (Fig. 1e) and shorter disease-free survival time (*P* = 0.013) of HCC patients (Fig. 1f) according to Kaplan–Meier analysis.

### PIM2 has strong oncogenic potential in HCC

**Fig. 2:**
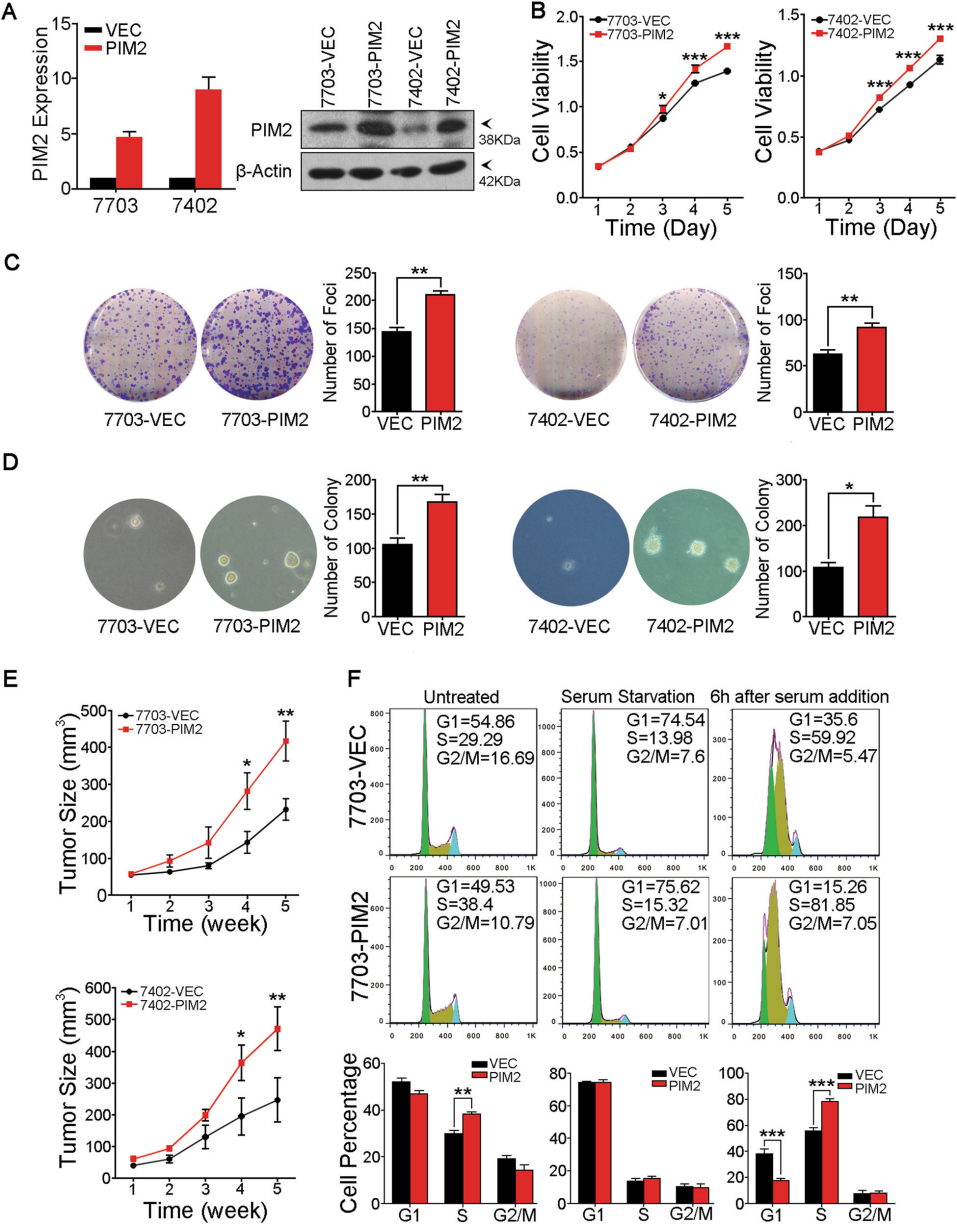
PIM2 overexpression enhanced HCC cells’ tumorigenic ability. **a** Overexpression of PIM2 in HCC cell lines 7703 and 7402 was confirmed by qRT-PCR and by Western Blot. **b** Growth curves of *PIM2*-transfected and empty vector-transfected cells was detected by XTT cell proliferation assay. The results are expressed as mean ± SD of three independent experiments. *p < 0.05, ***p < 0.001, independent Student’s *t* test. **c** Representative images of foci formed by *PIM2* and empty vector transduced cells in monolayer culture. Quantitative analysis of the number of foci are listed in the right panel. Results are presented as mean ± SD of three independent experiments. **p < 0.01, independent Student’s *t* test. **d** Representative images of colonies formed by *PIM2*-transduced and empty vector-transduced cells in soft agar assay. Quantitative analysis of the number of colonies are listed in the right panel. Results are presented as mean ± SD of three independent experiments. **p < 0.01, independent Student’s *t* test. **e** Representative images of tumors formed in nude mice following injection of empty vector-transduced (left dorsal flanks) and PIM2-transduced (right dorsal flanks) cells. The average tumor volume at each time point was expressed as mean ± SD in six nude mice. *p < 0.05, **p < 0.01, independent Student’s *t* test. **f** PIM2 overexpression promoted cycle progression. DNA content of 7703-VEC and 7703-PIM2 cells was analyzed by flow cytometry. Cell cycle synchronization was induced by treating cells with 400 μM l-Mimosine containing serum free medium for 24 h. To detect G1 to S phase transition, cells were stimulated with 10% FBS containing complete medium after cell cycle synchronization. Statistical results are expressed as mean ± SD of three independent experiments. **p < 0.01, ***p < 0.001, independent Student’s *t* test.

To characterize the function of *PIM2* in HCC, *PIM2* was stably transduced into QSG7703 and BEL7402 cell lines, which had relative lower expression levels of PIM2 (Fig. 1d). Empty vector-transduced cells were used as controls. Successful ectopic expression of PIM2 was determined by qRT-PCR and western blotting (Fig. 2a). Functional assays were performed to characterize the tumorigenic potential of PIM2. Cell growth assay showed that compared with empty vector-transduced cells, cell growth rates of *PIM2*-transduced cells were significantly higher (Fig. 2b). And compared to the empty vector-transduced cells, more foci were yielded in *PIM2*-transduced cells in the foci formation assay (Fig. 2c). *PIM2* overexpression also dramatically enhanced HCC cells’ anchorage-independent growth ability in soft agar (Fig. 2d).

To assess the ability of PIM2 in affecting tumorigenic potential of HCC cells in vivo, 5 × 10^6^ empty vector-transduced and *PIM2*-transduced cells were subcutaneously injected into the left and right dorsal flanks of six nude mice, respectively. Xenograft tumors formed by injected HCC cells was monitored every week for total 5 weeks, then the mice were sacrificed and xenograft tumors were collected for further study. Compared with empty vector controls, tumors formed by *PIM2*-transduced QSG7703 and BEL7402 were significantly larger (*P* < 0.05, student’s *t* test, Fig. 2e).

Since the functional studies showed that PIM2 was able to regulate HCC cell proliferation, we further performed cell cycle analysis to investigate if PIM2 was involved in the regulation of cell cycle progression of HCC cells. To induce cell cycle synchronization, 7703-VEC and 7703-PIM2 cells were treated with 400 nM l-Mimosine containing serum-free medium for 24 h. Then, serum-free medium was replaced with 10% fetal bovine serum (FBS) containing fresh complete medium, cells were collected at the indicated time points after serum stimulation and cell cycle analysis was performed by flow cytometry. Compared with 7703-VEC, 7703-PIM2 cells had a higher percentage of S stage when cultured in 10% FBS containing medium. After cell cycle synchronization, G1 and S phases reached the similar percentages between 7703-PIM2 and 7703-VEC cells. However, the percentage of cells in S phase was significantly higher in 7703-PIM2 cells than that in 7703-VEC cells 6 h after serum stimulation (Fig. 2f).

### PIM2 knockdown inhibited HCC cells’ tumorigenic ability

To further confirm *PIM2*’s tumorigenic potential, we designed two shRNAs to specifically knockdown the expression level of PIM2. Effective knockdown of PIM2 at both mRNA and protein levels was confirmed by qRT-PCR and western blotting (Supplementary Fig. S2A). PIM2 knockdown in HCC cells significantly slowed the cell proliferation rate (Supplementary Fig. S2B) and foci formation frequency (Supplementary Fig. S2C). PIM2 knockdown also dramatically attenuated HCC cells’ anchorage-independent growth ability in soft agar (Supplementary Fig. S2D).

To determine whether PIM2 knockdown could inhibit HCC cells’ tumorigenic ability in vivo, shGFP-transduced and shPIM2-transduced HCC cells were subcutaneously injected into the left and right dorsal flanks of nude mice (*n* = 6), respectively. Tumor volumes of PLC8024 cells injected mice were monitored for 5 weeks, and tumor volumes of Huh7 cells injected mice were monitored for 6 weeks. Tumors formed by shGFP-transduced cells were significantly larger than those formed by shPIM2-transduced cells for both PLC8024 and Huh7 (Supplementary Fig. S2E).

To further confirm PIM2’s role in regulating cell cycle progression, we performed cell cycle analysis on shGFP-transduced and shPIM2-transduced PLC8024 cells. The percentage of S stage cells was significantly reduced in 8024-shPIM2 cells compared with 8024-shGFP cells when cultured in 10% FBS containing medium (Supplementary Fig. S2F). Together, these results indicate that PIM2 could facilitate DNA synthesis and promote G1/S phase transition in HCC cells.

### PIM2 regulates HCC cells’ ability to tolerate chemotherapy

**Fig. 3:**
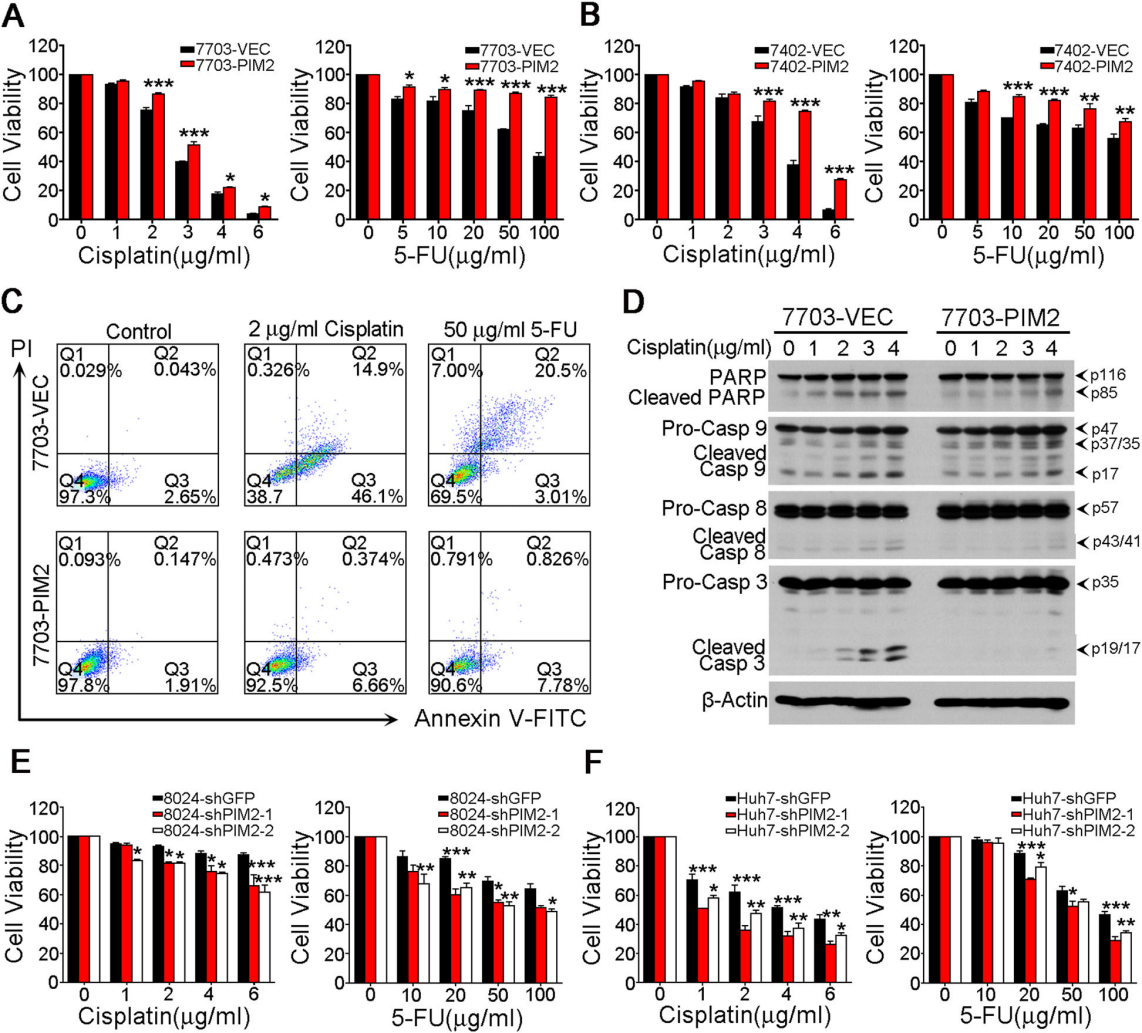
PIM2 overexpression enhanced HCC cells’ ability to tolerate chemotherapy. **a** Forty-eight hour after the treatment of Cisplatin or 5-FU at indicated concentrations, cell viability of empty vector-transduced or PIM2-transduced QSG7703 cells was detected by XTT assay. **b** Cell viability of Cisplatin and 5-FU treated empty vector-transduced or PIM2-transduced BEL7402 cells detected by XTT assay. **c** Apoptosis induced by Cisplatin (2 µg/ml) and 5-FU (50 µg/ml) was compared between 7703-VEC and 7703-PIM2 cells by flow cytometry analysis. Annexin-V staining positive cells were counted as apoptotic cells. **d** Activation of apoptosis-associated proteins (cleaved PARP, Caspase 9, Caspase 8, and Caspase 3) in 7703-VEC and 7703-PIM2 cells was analyzed by Western Blot after treatment with different concentrations of Cisplatin for 48 h. β-actin was used as a loading control. **e** Forty-eight hour after the treatment of Cisplatin or 5-FU at indicated concentrations, cell viability of shGFP-transduced or shPIM2-transduced PLC8024 cells was detected by XTT cell viability assay. **f** Cell viability of Cisplatin and 5-FU treated shGFP-transduced or shPIM2-transduced Huh7 cells was detected by XTT cell viability assay. Results are presented as mean ± SD of three independent experiments. *p < 0.05, **p < 0.01, ***p < 0.001, independent Student’s *t* test.

It has been reported that PIM2 could reverse growth factor depletion induced cell apoptosis through phosphorylating BAD^12^. In this study, we investigated whether PIM2 overexpression could enhance HCC cells’ ability to tolerate chemotherapy induced cell death. The survival index was measured by XTT cell survival assay. *PIM2*-transduced QSG7703 and BEL7402 cells have dramatically higher survival indexes than that of empty vector-transduced controls after exposure to different concentrations of Cisplatin or 5-FU for 48 h (Fig. 3a, b). Compared with 7703-VEC, flow cytometry analysis also showed a lower percentage of apoptotic cells in 7703-PIM2 treated by Cisplatin (2 µg/ml) and 5-FU (50 µg/ml) (Fig. 3c). Western Blot showed overexpression of PIM2 in 7703 cells attenuated the activation of apoptosis-associated proteins, such as cleaved PARP, Caspase 9, Caspase 8, and Caspase 3, stimulated by the treatment of different concentrations of Cisplatin (Fig. 3d). To further confirm PIM2’s function in protecting HCC cells from chemotherapy induced apoptosis, the survival index of shGFP-transduced and shPIM2-transducd HCC cells was compared by XTT cell survival assay. Forty-eight hour after different concentrations of Cisplatin or 5-FU treatment, the survival indexes of shPIM2-transduced cells were significantly lower than that of shGFP-transduced PLC8024 and Huh7 cells (Fig. 3e, f).

### PIM2 regulates metastatic ability of HCC cells

**Fig. 4:**
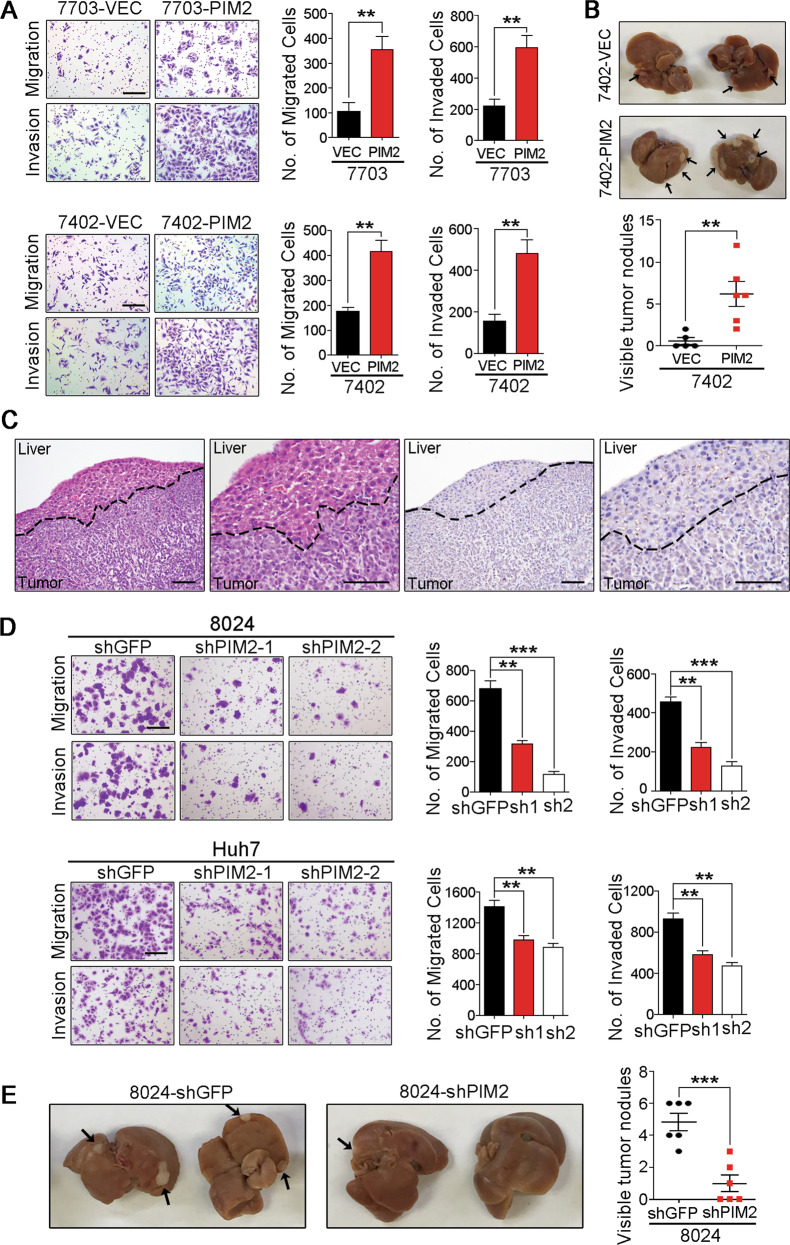
PIM2 regulates metastatic ability of HCC cells. **a** The cell migration rate and invasion rate of QSG7703 cells and BEL7402 cells transduced with empty vector or PIM2 was compared. Migrated or invaded cells was photographed under the ×10 objective lens, and the number of migrated or invaded cells was counted in five fields. Scale bar represents 200 µm. **p < 0.01, independent Student’s *t* test. **b** In vivo metastatic ability of empty vector-transduced and PIM2-transduced 7402 cells was evaluated by a spleen injection model, 6 weeks after cells injection into spleens of SCID mice, livers were collected and metastatic nodules on the liver were quantified. **p < 0.01, independent Student’s *t* test. **c** Metastatic tumors identified by H&E staining and IHC staining (the expression of PIM2 expression). Scale bar represents 100 µm. **d** The cell migration rate and invasion rate of 8024-shGFP and 8024-shPIM2 cells, as well as Huh7-shGFP and Huh7-shPIM2 were compared. **p < 0.01, ***p < 0.001, independent Student’s *t* test. **e** In vivo metastatic ability of shGFP-transduced and shPIM2-transduced 8024 cells was evaluated by a spleen injection model. ***p < 0.001, independent Student’s *t* test.

According to the clinicopathological analysis, PIM2 upregulation was significantly associated with vascular invasion and recurrence in HCC patients (Supplementary Table 1). Interestingly, the expression of PIM2 is higher in two highly metastatic cell lines H2M and 97H than that in H2P and 97L, which have lower metastatic ability (Fig. 1d). These indicate that PIM2 may be correlated with metastatic ability of HCC cells. In this study, the role of PIM2 in regulating metastatic potential of HCC cells was evaluated through in vitro migration and invasion assay, as well as in vivo metastasis assay. Compared with empty vector control, PIM2 overexpression significantly increased HCC cells’ migration and invasion ability of both QSG7703 and BEL7402 cells (Fig. 4a). More importantly, PIM2 overexpression dramatically enhanced BEL7402 cells’ ability to metastasize to the liver in an in vivo metastasis model (Fig. 4b). Metastatic tumors in the livers were confirmed by H&E staining and IHC staining (Fig. 4c). The role of PIM2 in regulating HCC cells’ metastatic ability was further confirmed by knockdown study. Compared with shGFP-transduced PLC8024 and Huh7 cells, shPIM2 transduction dramatically decreased their migration and invasion ability (Fig. 4d). Furthermore, PIM2 knockdown also decreased PLC8024 cell’s ability to metastasize to the liver (Fig. 4e).

### Ectopic expression of PIM2 activates NF-κB signaling pathway

**Fig. 5:**
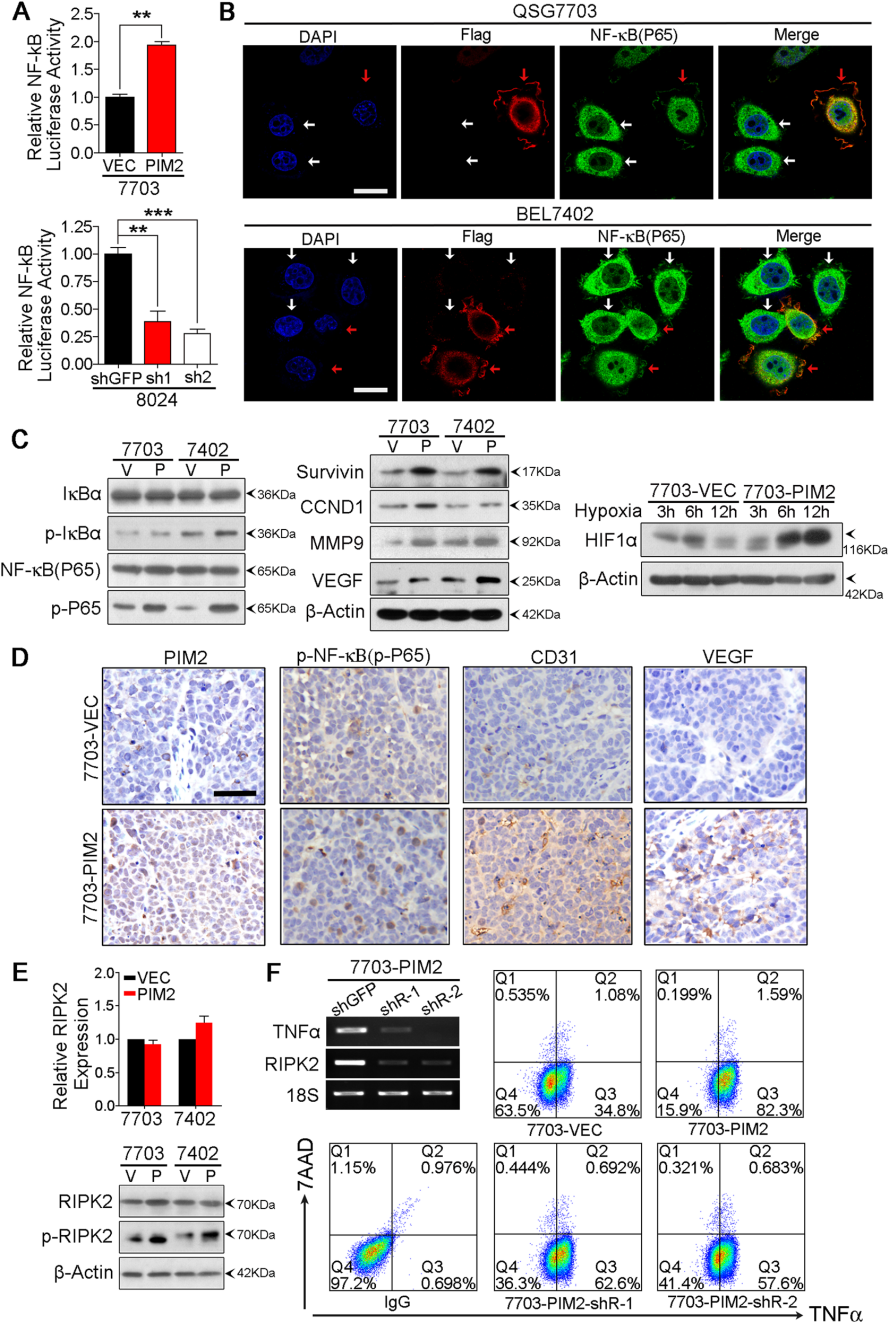
PIM2 overexpression activated NF-κB signaling pathway. **a** Relative NF-κB signaling pathway activity of empty vector-transfected and PIM2-transfected QSG7703 cells, and relative NF-κB signaling pathway activity of shGFP-transfected and shPIM2-transfected PLC8024 cells detected by luciferase reporter assay. The result is shown as mean ± SD of three independent experiments. **p < 0.01, ***p < 0.001, independent Student’s *t* test. **b** Immunofluorescence images of QSG7703 and BEL7402 cells transfected with Flag tagged PIM2 (indicated by red arrows) or their parental cells (indicated by white arrows). Cells were double stained with Flag antibody (red) and NF-κB (P65) antibody (green). Nuclei were labeled with DAPI (blue). Scale bar stands for 20 µm. **c** Expression of NF-κB (P65), p-NF-κB (p-P65), IκBα, p-IκBα, and NF-κB signaling pathway target genes, Survivin, CCND1, MMP9, VEGF, and HIF1α detected by Western Blot. β-Actin served as an internal control. V represents empty vector-transfected group and P represents PIM2-transfected group. **d** IHC images of paraffin sections from xenografts formed by 7703-VEC and 7703-PIM2 injection. Scale bar stands for 20 µm. **e** Relative expression level of RIPK2 in empty vector-transduced and PIM2-transduced 7703 and 7402 cells detected by qRT-PCR, and expression of RIPK2 and p-RIPK2 in empty vector-transduced and PIM2-transduced HCC cells detected by Western Blot. V represents empty vector-transduced group and P represents PIM2-transduced group. **f** Expression of RIPK2 and TNFα in shGFP-transduced and shRIPK2-transduced 7703-PIM2 cells detected by semiquantitative RT-PCR,18S rRNA was used as an internal control; and expression of TNFα in 7703-VEC, 7703-PIM2, 7703-PIM2-shRIPK2-1, and 7703-PIM2-shRIPK2-2 detected by flow cytometry.

Previous studies indicated that PIM2 was correlated with NF-κB signaling and pro-survival activity of PIM2 seemed to have been dependent on the activation of NF-κB signaling^15,16^. To detect whether PIM2 could regulate the activity of NF-κB signaling pathway, luciferase reporter assay was applied. PIM2 overexpression upregulated luciferase activity of NF-κB signaling pathway in QSG7703 cells (Fig. 5a). Accordingly, PIM2 knockdown downregulated luciferase activity of NF-κB signaling pathway in PLC8024 cells (Fig. 5a).

In canonical NF-κB signaling pathway, NF-κB normally locates in the cytoplasm through interacting with IκB, when stimulated by various activators, IκB was phosphorylated by upstream kinases and then degraded by proteasome. When separated with IκB, NF-κB undergo post-transcriptional modifications and translocates from cytoplasm to nuclei and functions as transcription regulators by interacting with other co-factors. Hundreds of genes that have κB binding sites will be transcribed as a result of the activation of NF-κB signaling pathway, and the transcription of these genes could further regulate various physiological processes^17^. Thus, a remarkable symbol of NF-κB signaling pathway activation is the trans-location of NF-κB (P65) from cytoplasm to nuclei. Here, Flag tagged *PIM2* was transiently transfected into HCC cells and double-label immunofluorescence was performed with anti-FLag and anti-NF-κB (P65) antibodies. We found that compared with nontransfected cells, in which NF-κB (P65) localized in the cytoplasm, transient expression of Flag tagged *PIM2* resulted in trans-location of NF-κB (P65) to the nuclei in both QSG7703 and BEL7402 cells (Fig. 5b). Western Blot analysis showed that PIM2 overexpression upregulated the expression of p-IκB and p-p65 as well as several targets of NF-κB signaling pathway, such as Survivin, CCND1, MMP9, VEGF, and HIF1α (Fig. 5c). A higher percentage of IHC staining of p-P65 was also observed in xenograft tumors formed by 7703-PIM2 than that formed by 7703-VEC cells (Fig. 5d). Abnormal angiogenesis is another symbol of NF-κB signaling pathway activation. In this study, we found xenograft tumors formed by 7703-PIM2 cells showed much more angiogenesis than those formed by 7703-VEC cells. IHC staining confirmed higher expression of CD31 and VEGF in xenograft tumors formed by 7703-PIM2 injection (Fig. 5d). Taken together, these data clearly indicate that PIM2 overexpression could directly activate NF-κB signaling pathway. Since *PIM2* encodes a serine/threonine kinase, the implementation of its biological functions largely depends on specific substrates phosphorylation. Given the existence of a conserved phosphorylation motif (RXXSXS) in receptor-interacting protein kinase (RIPK2) (Supplementary Fig. 3A)^18^, and RIPK2 was a well-known potent activator of NF-κB signaling pathway^19,20^, we hypothesized that the activation of NF-κB signaling pathway by PIM2 overexpression may depend on RIPK2 phosphorylation.

In this study, we found ectopic expression of PIM2 in HCC cells did not significantly affect mRNA level or protein expression level of RIPK2 (Fig. 5e), however, PIM2 overexpression upregulated the phosphorylation level of RIPK2 compared to PIM2 knockdown and control cells in vivo (Fig. 5e, Supplementary Fig. 3B). Furthermore, kinase-Glo Luminescent kinase assay results indicated that PIM2 could phosphorylate RIPK2 as the increasing amount of PIM2 led to the decrease of ATP in kinase reaction (Supplementary Fig. 3C). Enhanced level of phosphorylated RIPK2 could be detected in reactions with higher PIM2 concentrations (Supplementary Fig. 3D). Then, to elucidate whether RIPK2 was responsible for PIM2 overexpression induced NF-κB signaling pathway activation, we designed shRNAs to specifically target RIPK2 in PIM2 overexpressed QSG7703 cells. By applying the expression of TNFα to indicate the activation status of NF-κB signaling pathway, we found that RIPK2 knockdown decreased the expression level of TNFα, which was confirmed by semiquantitative RT-PCR (Fig. 5f) and flow cytometry analysis (Fig. 5f). These results suggested that PIM2 overexpression induced NF-κB signaling pathway activation may depend on upregulation of phosphorylated RIPK2.

### A positive-feedback loop between PIM2 and TNFα

**Fig. 6:**
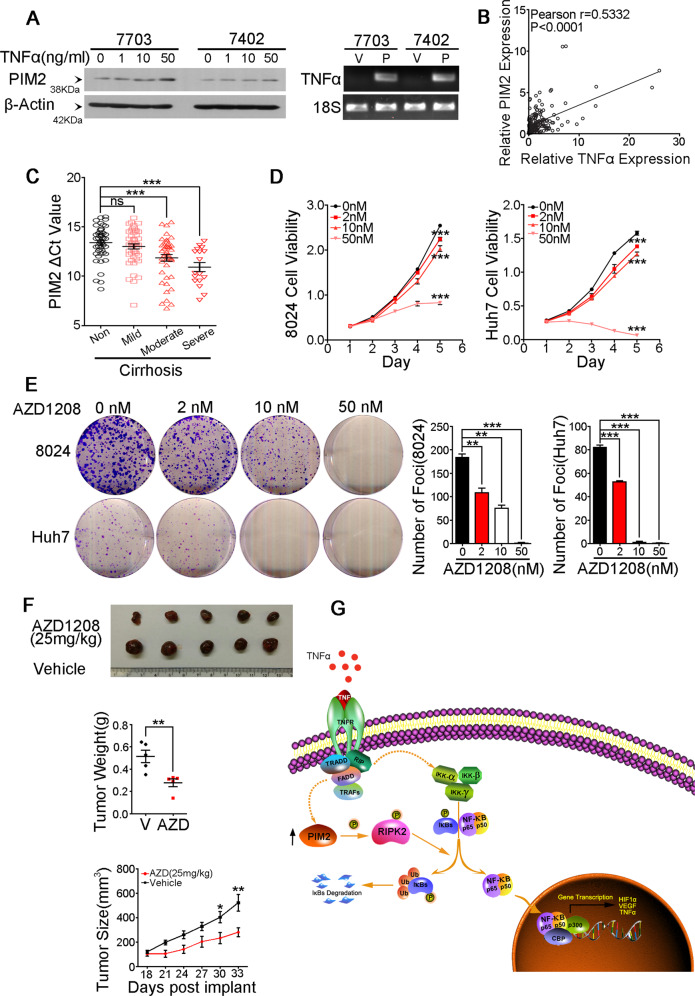
A positive-feedback loop between PIM2 and TNFα. **a** The expression of PIM2 in different concentration of TNFα treated HCC cells detected by Western Blot, β-Actin served as an internal control. Expression of TNFα in empty vector-transduced and PIM2-transduced HCC cells detected by semiquantitative RT-PCR. V represents empty vector-transduced group and P represents PIM2-transduced group. **b** Relative expression level of PIM2 and TNFα was detected in 92 pairs of HCC tumor and nontumor samples by qRT-PCR. **c** Average ΔCt value of PIM2 in HCC patients without cirrhosis and with different degree of cirrhosis. ***p < 0.001, independent Student’s *t* test. **d** AZD1208 treatment decreased growth rates of 8024 and Huh7 cells. ***p < 0.001, independent Student’s *t* test. **e** AZD1208 treatment decreased PLC8024 and Huh7’s ability to form foci in monolayer culture in a dose dependent manner. **p < 0.01, ***p < 0.001, independent Student’s *t* test. **f** Eighteen days after injection of PLC8024 cells in nude mice, oral administration of 25 mg/kg AZD1208 or vehicle control once 2 days was performed, tumors were monitored once every three days for total 15 days. *n* = 5 per group, *p < 0.05, **p < 0.01, independent Student’s *t* test. **g** A schematic diagram illustrating the proposed TNFα/PIM2/NF-κB Signaling Axis in HCC progression.

In this study, upregulation of PIM2 was observed in more than 50% of HCC patients, we tried to investigate how the upregulation of PIM2 was achieved. Based on previous studies, the expression of PIM2 can be regulated by various cytokines, growth factors, and chemokines. Among these factors, we were particularly interested on TNFα, since most HCC patients in our study had chronic hepatitis history and TNFα is a well-known pro-inflammatory cytokine. Similar with previous report on lymphoma cells, TNFα treatment could upregulate the expression of PIM2 in a dose dependent manner on both QSG7703 and BEL7402 cells (Fig. 6a). Interestingly, we found that PIM2 overexpression could in turn upregulate the expression of TNFα (Fig. 6a). The correlation between the expression levels of PIM2 and TNFα was further investigated in HCC clinical samples by qRT-PCR. The result revealed a dramatic correlation between gene expression level of TNFα and PIM2 in HCC patients (Fig. 6b). These results demonstrated the existence of a positive correlation between PIM2 and TNFα, suggesting a feedback loop between these two factors. Interestingly, we found that compared with non-cirrhotic HCC patients, those with liver cirrhosis had a lower average ΔCt value of PIM2, which indicated a higher expression level of PIM2 in cirrhotic HCC patients. And we further divided cirrhotic HCC patients into mild, moderate, and severe group according to the severity of cirrhosis, and found that the average ΔCt value of PIM2 was gradually decreased as the severity of cirrhosis increased (Fig. 6c). These results indicate that PIM2’s expression level positively correlated with severity of liver cirrhosis in HCC patients.

### PIM kinase inhibitor inhibits HCC cells’ tumorigenic ability both in vitro and in vivo

AZD1208, a potent inhibitor of PIM kinase, has been reported effective in attenuating tumorigenic ability of many human malignancies, including non-Hodgkin lymphoma^21^, acute myeloid leukemia^22^, and prostate cancer^23^. In this study, we tested whether AZD1208 was effective in impairing HCC cells’ tumorigenic ability. Compared with vehicle control, AZD1208 treatment dramatically decreased 8024 and Huh7 cells’ growth rates (Fig. 6d) and their abilities to form foci in monolayer culture in a dose-dependent manner (Fig. 6e). More importantly, compared with vehicle control, 25 mg/kg AZD1208 treatment significantly inhibited the growth of xenograft tumors in nude mice (Fig. 6f). These results suggested that PIM kinase inhibition may serve as a therapeutic target in the treatment of HCC.

## Discussion

Up to 20% human malignancies are developed from chronic inflammation, HCC is the case. Hepatitis B and/or C virus infection caused chronic inflammation and liver cirrhosis is the most important risk factor for developing HCC. It has been demonstrated that TNFα and IL-6 played pivotal roles in inflammation induced HCC incidence^9^ and multiple studies showed that the expression of PIM2 could be regulated by various growth factors and cell cytokines^10–12^. For example, the expression of PIM2 in multiple myeloma cells could be regulated by IL-6 and TNF family cytokines, TNFα, BAFF, and APRIL. Interestingly, IL-6 could cooperate with TNFα in inducing upregulation of PIM2 expression^10^. These studies indicate that PIM2 may play an important role in inflammation induced carcinogenesis.

Abnormal expression of PIM kinases had been observed in many kinds of human cancers. Previous studies on PIM2 indicated that it played multiple roles in regulating malignant phenotypes of cancer cells. For example, PIM2 could inhibit apoptosis of cancer cells through activating API-5^24^, or through phosphorylating BAD on Serine 112^25^; PIM2 is required for cell growth and proliferation of multiple myeloma^26,27^; and PIM2 was found to be upregulated by the treatment of cisplatin and limited its efficacy in ovarian cancer cells^28^. In this study, we found that up-regulation of PIM2 played multiple roles in the development and progression of HCC. Generally, PIM2 up-regulation enhanced HCC cells’ ability to proliferate and to tolerate apoptosis through elevating the expression of CCND1 and Survivin, these are important for the initial stage of HCC tumorigenesis; PIM2 overexpression upregulated HIF1α and VEGF and enhanced angiogenesis, a rate limiting factor of HCC progression, which is critical for the progression of HCC; furthermore, PIM2 upregulation could elevate the expression of MMP9 and enhance HCC cells’ metastatic ability, which is pivotal for the development of advanced stage HCC.

The relationship between PIM2 and NF-κB signaling pathway was obscure, although there was report about simultaneous alteration of PIM2 and NF-κB genes’ expression levels^24,29^. Some studies indicated that PIM2 was a downstream target of NF-κB signaling^30^, while others showed that PIM2 worked upstream of NF-κB signaling^31^. Here, we found that the expression of PIM2 in HCC cells could be upregulated by the stimulation of TNFα, and ectopic expression of PIM2 in HCC cells could in turn upregulate the expression of TNFα. This indicates the existence of a feedback loop between PIM2 and NF-κB signaling. Notably, it has been reported that a positive feedback loop between NF-κB and TNFα promoted acute myeloid leukemia-initiating cell capability^32^. These kinds of feedback loops between NF-κB signaling and its upstream stimulators or downstream targets maybe ubiquitously existed, and played important roles in tumor initiation and progression of various kinds of cancers. In this study, we identified RIPK2 as a potential target responsible for PIM2 overexpression induced NF-κB signaling pathway activation. Besides p-RIPK2, upregulation of p-FAK, p-MEK1/2 and p-ERK were also found in PIM2 transfected HCC cells (data not shown). As a serine/threonine kinase, PIM2 may exert extensive biological functions through phosphorylating many kinds of specific substrates, which ultimately induced malignant phenotypes of HCC (Fig. 6g).

## Conclusion

Taken together, our study revealed a novel mechanism underlying HCC incidence and progression, and this is very likely an early event in inflammation induced HCC tumorigenesis. Apart from the oncogenic function of PIM2 in HCC, we found that the expression level of PIM2 correlates with severity of liver cirrhosis in HCC patients. This indicates that the feedback loop between PIM2 and TNFα maybe a driven force between chronic liver inflammation and cirrhosis. It will be interesting to study the role of PIM2 in the progression of liver cirrhosis on transgenic mouse in the future. More importantly, PIM kinase inhibitor showed effective in attenuating HCC cells’ tumorigenic ability, and PIM kinases may serve as novel therapeutic targets in HCC patients.

## Supplementary information

Supplementary materials

Supplementary Figure 1. PIM2 expression in HCC clinical samples.

Supplementary Figure 2. PIM2 knockdown attenuated HCC cells’ tumorigenic ability.

Supplementary Figure 3. PIM2 can phosphorylate RIPK2 in vitro and vivo.

## References

[1] el-Serag, H. B. Epidemiology of hepatocellular carcinoma. *Clin. Liver Dis.* **5**, 87–107, vi (2001). 11218921 10.1016/s1089-3261(05)70155-0

[2] Badvie, S. Hepatocellular carcinoma. *Postgrad. Med. J.* **76**, 4–11 (2000). 10622772 10.1136/pmj.76.891.4 PMC1741466

[3] El-Serag, H. B. Epidemiology of viral hepatitis and hepatocellular carcinoma. *Gastroenterology* **142**, 1264–1273, e1261 (2012). 22537432 10.1053/j.gastro.2011.12.061 PMC3338949

[4] Ma, N. F. et al. Isolation and characterization of a novel oncogene, amplified in liver cancer 1, within a commonly amplified region at 1q21 in hepatocellular carcinoma. *Hepatology* **47**, 503–510 (2008). 18023026 10.1002/hep.22072

[5] Li, Y. et al. SPOCK1 is regulated by CHD1L and blocks apoptosis and promotes HCC cell invasiveness and metastasis in mice. *Gastroenterology* **144**, 179–191, e174 (2013). 23022495 10.1053/j.gastro.2012.09.042

[6] Fu, L. et al. Down-regulation of tyrosine aminotransferase at a frequently deleted region 16q22 contributes to the pathogenesis of hepatocellular carcinoma. *Hepatology* **51**, 1624–1634 (2010). 20209601 10.1002/hep.23540

[7] Liu, M. et al. Allele-specific imbalance of oxidative stress-induced growth inhibitor 1 associates with progression of hepatocellular carcinoma. *Gastroenterology* **146**, 1084–1096 (2014). 24417816 10.1053/j.gastro.2013.12.041

[8] Chen, L. et al. Recoding RNA editing of AZIN1 predisposes to hepatocellular carcinoma. *Nat. Med.* **19**, 209–216 (2013). 23291631 10.1038/nm.3043 PMC3783260

[9] Park, E. J. et al. Dietary and genetic obesity promote liver inflammation and tumorigenesis by enhancing IL-6 and TNF expression. *Cell* **140**, 197–208 (2010). 20141834 10.1016/j.cell.2009.12.052 PMC2836922

[10] Asano, J. et al. The serine/threonine kinase Pim-2 is a novel anti-apoptotic mediator in myeloma cells. *Leukemia* **25**, 1182–1188 (2011). 21475253 10.1038/leu.2011.60

[11] Hiasa, M. et al. Pim-2 kinase is an important target of treatment for tumor progression and bone loss in myeloma. *Leukemia* **29**, 207–217 (2015). 24787487 10.1038/leu.2014.147

[12] Fox, C. J. et al. The serine/threonine kinase Pim-2 is a transcriptionally regulated apoptotic inhibitor. *Genes Dev.* **17**, 1841–1854 (2003). 12869584 10.1101/gad.1105003 PMC196230

[13] Cuypers, H. T. et al. Murine leukemia virus-induced T-cell lymphomagenesis: integration of proviruses in a distinct chromosomal region. *Cell* **37**, 141–150 (1984). 6327049 10.1016/0092-8674(84)90309-x

[14] Liu, L. et al. Maelstrom promotes hepatocellular carcinoma metastasis by inducing epithelial-mesenchymal transition by way of Akt/GSK-3beta/Snail signaling. *Hepatology* **59**, 531–543 (2014). 23929794 10.1002/hep.26677

[15] Fox, C. J., Hammerman, P. S. & Thompson, C. B. The Pim kinases control rapamycin-resistant T cell survival and activation. *J. Exp. Med.* **201**, 259–266 (2005). 15642745 10.1084/jem.20042020 PMC2212793

[16] Hammerman, P. S., Fox, C. J., Birnbaum, M. J. & Thompson, C. B. Pim and Akt oncogenes are independent regulators of hematopoietic cell growth and survival. *Blood* **105**, 4477–4483 (2005). 15705789 10.1182/blood-2004-09-3706 PMC1895036

[17] Karin, M. Nuclear factor-kappaB in cancer development and progression. *Nature* **441**, 431–436 (2006). 16724054 10.1038/nature04870

[18] Hu, J. et al. PhosphoNetworks: a database for human phosphorylation networks. *Bioinformatics* **30**, 141–142 (2014). 24227675 10.1093/bioinformatics/btt627 PMC3866559

[19] McCarthy, J. V., Ni, J. & Dixit, V. M. RIP2 is a novel NF-kappaB-activating and cell death-inducing kinase. *J. Biol. Chem.* **273**, 16968–16975 (1998). 9642260 10.1074/jbc.273.27.16968

[20] Dorsch, M. et al. Identification of a regulatory autophosphorylation site in the serine-threonine kinase RIP2. *Cell. Signal.* **18**, 2223–2229 (2006). 16824733 10.1016/j.cellsig.2006.05.005

[21] Kreuz, S., Holmes, K. B., Tooze, R. M. & Lefevre, P. F. Loss of PIM2 enhances the anti-proliferative effect of the pan-PIM kinase inhibitor AZD1208 in non-Hodgkin lymphomas. *Mol. Cancer* **14**, 205 (2015). 26643319 10.1186/s12943-015-0477-z PMC4672512

[22] Keeton, E. K. et al. AZD1208, a potent and selective pan-Pim kinase inhibitor, demonstrates efficacy in preclinical models of acute myeloid leukemia. *Blood* **123**, 905–913 (2014). 24363397 10.1182/blood-2013-04-495366 PMC3916880

[23] Kirschner, A. N. et al. PIM kinase inhibitor AZD1208 for treatment of MYC-driven prostate cancer. *J. Natl Cancer Inst.*10.1093/jnci/dju407 (2015). 10.1093/jnci/dju407 PMC4326311 25505253

[24] Ren, K., Zhang, W., Shi, Y. & Gong, J. Pim-2 activates API-5 to inhibit the apoptosis of hepatocellular carcinoma cells through NF-kappaB pathway. *Pathol. Oncol. Res.* **16**, 229–237 (2010). 19821157 10.1007/s12253-009-9215-4

[25] Yan, B. et al. The PIM-2 kinase phosphorylates BAD on serine 112 and reverses BAD-induced cell death. *J. Biol. Chem.* **278**, 45358–45367 (2003). 12954615 10.1074/jbc.M307933200

[26] Lu, J. et al. Pim2 is required for maintaining multiple myeloma cell growth through modulating TSC2 phosphorylation. *Blood* **122**, 1610–1620 (2013). 23818547 10.1182/blood-2013-01-481457 PMC3953014

[27] Lu, J. et al. Pim2 is required for maintaining Multiple Myeloma cell proliferation through modulating mTORC1 pathway. *Cancer Res.*10.1158/1538-7445.AM2013-5194 (2013).

[28] Musiani, D. et al. PIM2 kinase is induced by cisplatin in ovarian cancer cells and limits drug efficacy. *J. Proteome Res.* **13**, 4970–4982 (2014). 25099161 10.1021/pr500651n

[29] Kapelko-Slowik, K. et al. Expression of PIM-2 and NF-kappaB genes is increased in patients with acute myeloid leukemia (AML) and acute lymphoblastic leukemia (ALL) and is associated with complete remission rate and overall survival. *Postepy Hig. Med. Dosw.* **67**, 553–559 (2013). 10.5604/17322693.1052449 23752607

[30] Bansal, K. et al. PIM2 induced COX-2 and MMP-9 expression in macrophages requires PI3K and Notch1 signaling. *PLoS ONE* **4**, e4911 (2009). 19290049 10.1371/journal.pone.0004911 PMC2654112

[31] Hammerman, P. S. et al. Lymphocyte transformation by Pim-2 is dependent on nuclear factor-kappaB activation. *Cancer Res.* **64**, 8341–8348 (2004). 15548703 10.1158/0008-5472.CAN-04-2284

[32] Kagoya, Y. et al. Positive feedback between NF-kappaB and TNF-alpha promotes leukemia-initiating cell capacity. *J. Clin. Investig.* **124**, 528–542 (2014). 24382349 10.1172/JCI68101 PMC3904603

