# Supplementary material for: PIM2 promotes hepatocellular carcinoma tumorigenesis and progression through activating NF-κB signaling pathway
Source: Cell Death Dis. 2020 Jul 2;11(7):510. doi: 10.1038/s41419-020-2700-0 (PMC7343807; doi:10.1038/s41419-020-2700-0)
Supplement: Supplementary file 1 — Supplementary materials [file 41419_2020_2700_MOESM1_ESM.docx]

**Supplementary**

**Supplementary Materials and Methods**

***HCC Clinical Samples and Cell Lines***. HCC specimens (tumor and paired adjacent non-tumor tissues) were obtained from patients who underwent hepatectomy from HCC at Sun Yat-Sen University Cancer Center (Guangzhou, China). All HCC patients gave written informed consent on the use of clinical specimens for medical research. The samples used in this study were approved by the Committees for Ethical Review of Research Involving Human Subjects at the Sun Yat-Sen University Cancer Center. Two immortalized hepatocyte cell lines (MiHA and LO2) and 11 HCC cell lines (QSG7701, QSG7703, BEL7402, PLC8024, Huh7, HepG2, Hep3B, H2P, H2M, 97H and 97L) were used in this study. All cell lines used in this study were tested for absence of mycoplasma contamination and authenticated by morphological observation (MycoAlert; Lonza, Rockland, ME) 3 months ago. Moreover, cell sorting by flow cytometry was performed on HCC cells using phycoerythrin-conjugated monoclonal mouse

anti-human CD133/1 (AC133; Miltenyi Biotec, Auburn, CA) to check CD133 expression. Expression

of CD133 is consistent with previous studies (*Ma S, Chan KW, et al. Gastroenterology 2007;132:2542-2556*).

***Plasmid Constructs and Lenti-virus Transduction.*** Full-length of human *PIM2* gene was PCR amplified and cloned into pLenti6/v5-D-topo expression vector (Invitrogen) according to manufacturer’s instructions. *PIM2* containing lenti-virus was then stably transduced into HCC cell lines QSG7703 and BEL7402 by blasticidin selection (2µg/mL). Empty vector transduced cells were used as controls. Two short hairpin RNAs (shRNA) specifically targeting on *PIM2*: 5′- GATGAACCCTACACTGACTTT-3′ and 5′-GCCCAGGATCTCTTTGACTAT-3′ and two shRNAs specifically targeting on *RIPK2*: 5′-GTTACTTGTCTAAGATGCAAT-3′ and 5′-GCACAATATGACTCCTCCTTT-3′ were cloned into pLL3.7 lenti-viral vector. PLC8024 and Huh7 were transduced with shRNAs to establish stable *PIM2* knockdown cell lines. Cells transduced with shGFP: 5′-ACAACAGCCACAACGTCTATA-3′ were used as negative controls.

***Immunofluorescence (IF) Staining and Confocal Microscopy.*** Cells were transiently transfected with Flag tagged PIM2, and 48hr later, cells were fixed with 4% PFA at room temperature for 15min, permeabilized and blocked with 0.1% PBS-T in 5% BSA. Primary antibodies: Flag (1:100, Sigma, F1804) and NF-κB (1:100, Santa Cruz, Sc-372) were incubated at 4℃ overnight, then cells were thoroughly washed by PBS and followed by incubation with secondary antibodies. The nuclei was stained with DAPI (Invitrogen, CA).Images were captured using a confocal laser scanning microscope (Zeiss LSM510 META).

***Functional Assays.*** For cell proliferation assay, cells were seeded in 96-well plates at a density of 1,000 cells per well, and cell growth rate was detected by the XTT kit (Roche Diagnostics). For foci formation assay, 1,000 cells were seeded in 6-well plates and cultured for 2 weeks, cell colonies were fixed with 4% PFA and counted by crystal violet staining. For colony formation assay, 10,000 cells were seeded in 0.3% soft agar containing medium for 3 weeks, colonies were counted under microscopy. The results are expressed as the mean ±SD of three independent experiments. Xenograft tumor formation assay was established by subcutaneous injection of equal number of empty vector-transduced or shGFP-transduced cells and PIM2 transduced or shPIM2-transduced cells into the left and right dorsal flank of 4-week-old nude mice, respectively. Tumor formation in nude mice was checked every week for total 5~6 weeks. All mice were randomized to experiment and control groups. For animal studies, no blinding was done. All animal experiments were performed under the institutional standard guidelines at The University of Hong Kong.

***RNA Extraction and qRT-PCR.*** Total RNA was extracted using the TRIZOL Reagent (Invitrogen) and reverse transcription was performed using the Super-script III Reverse Transcriptase (Invitrogen). The cDNA was subjected to quantitative real-time PCR (qRT-PCR) using the SYBR Green PCR Kit (Applied Biosystems, Carlsbad, CA). The relative levels of expression were quantified and analyzed using SDS 2.3 software (Applied Biosystems, FosterCity, CA). The relative expression level (defined as fold change) of PIM2 (2^-ΔΔCt^) was normalized to the endogenous 18S rRNA reference (ΔCt) and related to the amount of target gene in control sample, which was defined as the calibrator at 1.0. Three independent experiments were performed to analyze the relative gene expression and each sample was tested in triplicate. qRT-PCR Primers were listed as below.

PIM2-Forward:5′-ACTGACTTTGATGGGACAAGGG-3′ and

PIM2- Reverse: 5′-AATGTCCCCACACACCATGT-3′;

18S-Forward:5′-CTCTTAGCTGAGTGTCCCGC-3′ and

18S-Reverse: 5′-CTGATCGTCTTCGAACCTCC-3′;

TNFα-Forward:5′-AGGCAGTCAGATCATCTTCTCG-3′ and

TNFα-Reverse: 5′-ATGAGGTACAGGCCCTCTGAT-3′; RIPK2-Forward:5′-GCTCGACAGTGAAAGAAAGGATG-3′ and

RIPK2-Reverse:5′-CAATGGCCAAGCAACATCAGG-3′

**Western Blot Antibodies:**PIM2 (1:1,000, Abgent, Ap7933a), Caspase 3 (1:1,000, Cell Signaling Technology, 9662), Caspase 8 (1:1,000, Cell Signaling Technology, 9746), Caspase 9 (1:1,000, Cell Signaling Technology, 9502), PARP (1:1,000, Cell Signaling Technology, 9542), HIF1α (1:1,000, GeneTex, GTX127309), β-Actin (1:5,000, Abcam, Ab6276), Survivin (1:1,000, Boster, BA1420), CCND1 (1:1,000, Cell Signaling Technology, 2926), MMP9 (1:1,000, Abcam, Ab38898), VEGF (1:1,000, Boster, BA0407), p-RIPK2(1:1000, Thermo Fisher, PA5104447), RIPK2 (1:1000, Abclonal, A2498), NF-κB p65 (1:1,000, Cell Signaling Technology, 8242), Phospho-NF-κB p65 (Ser536) (1:1,000, Cell Signaling Technology, 3033), IκBα(1:1,000, Cell Signaling Technology, 4814), Phospho-IκBα (Ser32) (1:1,000, Cell Signaling Technology, 2859), Anti-rabbit IgG, HRP-linked (1:10,000, Cell Signaling Technology, 7074), Anti-mouse IgG, HRP-linked (1:10,000, Cell Signaling Technology, 7076).

**IHC antibodies:**PIM2 (1:100, Abgent, Ap7933a), CD31 (1:100, Abcam, ab28364), VEGF (1:50, Dako, M727329), p-NF-κB p65 (Ser 536): (1:100, Santa Cruz, sc-33020).

***Migration and Invasion Assays.*** Migration assay and invasion assay was performed with Falcon® Cell Culture Inserts (Corning) with 8.0μm PET membrane and BD BioCoat Matrigel Invasion Chambers, respectively, following the manufacturer’s instructions. The migrated cells were stained with 1% crystal violet and counted under a microscope. Both experiments were repeated in triplicate independently.

***Experimental Metastasis Assay.*** For the overexpression study, two groups of 6 SCID mice each were given spleen injections of 1×10^6^ 7402-VEC cells and 7402-PIM2 cells, respectively. For the knockdown study, two groups of 6 SCID mice each were given spleen injections of 1×10^6^ 8024-shGFP cells and 8024-shPIM2 cells, respectively. 6 weeks later, the mice were sacrificed and the tumor nodules formed on the surfaces of livers were counted. Livers were excised and embedded in paraffin for H&E and IHC study. All mice were randomized to experiment and control groups. For animal studies, no blinding was done.

***Kinase Assays.*** In *vitro* kinase assays were performed using Kinase-Glo Luminescent kinase assay (Promega Corporation, Madison, WI, USA) and western blotting with recombinant PIM2 and RIPK2 protein (Thermo Fisher, PA3649 and PA4213). In *vivo* kinase assays were performed using 8024 and Huh7 cells transfected with both Flag-RIPK2 and either PIM2 or shPIM2. After 48 hours, transient transfected cells were washed and then lysates were collected for immunoprecipitation.

***Statistical Analysis.*** Statistical analysis was performed by IBM SPSS Statistics 19 software (SPSS Inc., Chicago, IL). The association of PIM2 up-regulation in HCC patients with different clinical-pathological features was analyzed by two-tailed Chi-square test and survival analysis were assessed by Kaplan-Meier plots and log-rank tests. Independent Student’s *t*-test was applied to assess the statistical significance between two pre-selected groups. Statistical significance was declared if *P* <0.05.

**Table S1. Clinicopathological Correlation of PIM2 Expression in 134 Primary HCCs**

| Features | Total | PIM2 | | *p* value |
| --- | --- | --- | --- | --- |
|  |  | Normal Expression | Up-regulation |  |
| Sex |  |  |  |  |
| Male | 115 | 50 (37.3%) | 65 (48.5%) | 0.242 |
| Female | 19 | 11 (8.2%) | 8 (6.0%) |  |
| Age, y |  |  |  |  |
| <60 | 107 | 51 (38.1%) | 56 (41.8%) | 0.322 |
| ≥60 | 27 | 10 (7.5%) | 17 (12.6%) |  |
| Hepatitis B surface antigen* |  |  |  |  |
| Negative | 28 | 13 (9.8%) | 15(11.4%) | 0.836 |
| Positive | 104 | 46 (34.8%) | 58(43.9%) |  |
| Serum AFP (ng/mL)* |  |  |  |  |
| <400 | 71 | 28 (21.2%) | 43 (32.6%) | 0.190 |
| ≥400 | 61 | 31 (23.5 %) | 30 (22.7%) |  |
| Tumor size, cm*^†^ |  |  |  |  |
| <5 | 40 | 17 (13.0%) | 23 (17.5%) | 0.786 |
| ≥5 | 91 | 41 (31.3%) | 50 (38.2%) |  |
| Cirrhosis* |  |  |  |  |
| Absent | 46 | 20 (15.2%) | 26 (19.7%) | 0.837 |
| Present | 86 | 39 (29.5%) | 47 (35.6%) |  |
| Vascular invasion*^‡^ |  |  |  |  |
| Absent | 119 | 57 (43.2%) | 62 (47%) | **0.025** |
| Present | 13 | 2 (1.5%) | 11 (8.3%) |  |
| Capsule* |  |  |  |  |
| Complete | 45 | 22 (16.7%) | 23 (17.4%) | 0.486 |
| No/incomplete | 87 | 37 (28.0%) | 50 (37.9%) |  |
| Differentiation* |  |  |  |  |
| Well/moderate | 54 | 21 (16.3%) | 33 (25.6%) | 0.304 |
| Poor | 75 | 36 (27.9%) | 39 (30.2%) |  |
| Recurrence* |  |  |  |  |
| Absent | 56 | 32 (24.2%) | 24 (18.2%) | **0.014** |
| Present | 76 | 27 (20.4%) | 49 (37.2%) |  |
| TNM Stage (AJCC)^#^ |  |  |  |  |
| I | 59 | 34 (25.4%) | 25 (18.7%) | **0.013** |
| II/III | 75 | 27 (20.1%) | 48 (35.8 %) |  |

Significant differences are shown in bold. *Partial data not available; statistics based on available data.^†^Tumor size was measured by the length of the largest tumor nodule.^‡^Defined by findings on final pathological analysis (microscopic and major).^#^American Joint Committee on Cancer classification.

**Supporting Figure Legends:**

**Supplementary Figure 1. PIM2 expression in HCC clinical samples.** PIM2 expression in 16 pairs of randomly selected HCC tumor and matched adjacent non-tumor tissues detected by Western Blot. GAPDH was used as a loading control.

**Supplementary Figure 2. PIM2 knockdown attenuated HCC cells’ tumorigenic ability.** **(A)** Two shRNAs that can specifically knockdown PIM2 in HCC cell lines PLC8024 and Huh7 detected by qRT-PCR and by Western Blot. **(B)** Growth curves of shGFP-transduced and shPIM2-transduced cells were detected by XTT cell proliferation assay. The results are presented as mean ± SD of three independent experiments. ***p<0.001, independent Student’s t test. **(C)** Representative images of foci formed by shGFP-transduced and shPIM2-transduced cells in monolayer culture. Quantitative analyses of the number of foci are listed in the right panel. Results are presented as mean ± SD of three independent experiments. *p<0.05, **p<0.01, independent Student’s t test. **(D)** Representative images of colonies formed by shGFP-transduced and shPIM2-transduced cells in soft agar assay. Quantitative analyses of the number of colonies are listed in the right panel. Results are presented as mean ± SD of three independent experiments. **p<0.01, independent Student’s t test. **(E)** Representative images of tumors formed in nude mice by subcutaneous injection of shGFP-transduced and shPIM2-transduced cells. The average tumor volume at each time point was expressed as mean ± SD in 6 nude mice. **p<0.01, ***p<0.001, independent Student’s t test. **(F)** PIM2 knockdown arrested cell cycle progression. DNA content of 8024-shGFP and 8024-shPIM2 cells was analyzed by flow cytometry. Cell cycle synchronization was induced by treating cells with L-Mimosine (400μM) in serum free medium. To detect G1 to S phase transition, cells were stimulated with complete medium (10% FBS containing) for 12hr after cell cycle synchronization.

**Supplementary Figure 3.** PIM2 can phosphorylate RIPK2 in *vitro* and *vivo*. **(A)**. the predicted phosphorylation motif of PIM2. **(B)** In *vivo* kinase assays were performed using 8024 and Huh7 cells transfected with both Flag-RIPK2 and either PIM2 or shPIM2. RIPK2 was immunoprecipitated with FLAG beads. p-RIPK2 and RIPK2 were detected immunoprecipitated product. Anti-Flag western blot is shown as a loading control. **(C)**. PIM2 kinase and RIPK2 combination was confirmed with Promega’s kinase-Glo^TM^ Assays. A linear relationship between the luminescent signal and the amount of ATP in the reaction buffer from 0-10µM (Upper). Kinase reactions containing 40mM Tris-HCl (pH7.5), 0.1mg/ml BSA, 20mM MgCl_2_ were performed at 10µM ATP, 4 pmol RIPK2 with different amount of PIM2 for 30 minutes (Down). **(D)** Enhanced level of phosphorylated RIPK2 was observed in the reaction with higher PIM2 concentrations.
