# Supplementary figures and images for: PIM2 promotes hepatocellular carcinoma tumorigenesis and progression through activating NF-κB signaling pathway
Source: Cell Death Dis. 2020 Jul 2;11(7):510. doi: 10.1038/s41419-020-2700-0 (PMC7343807; doi:10.1038/s41419-020-2700-0)

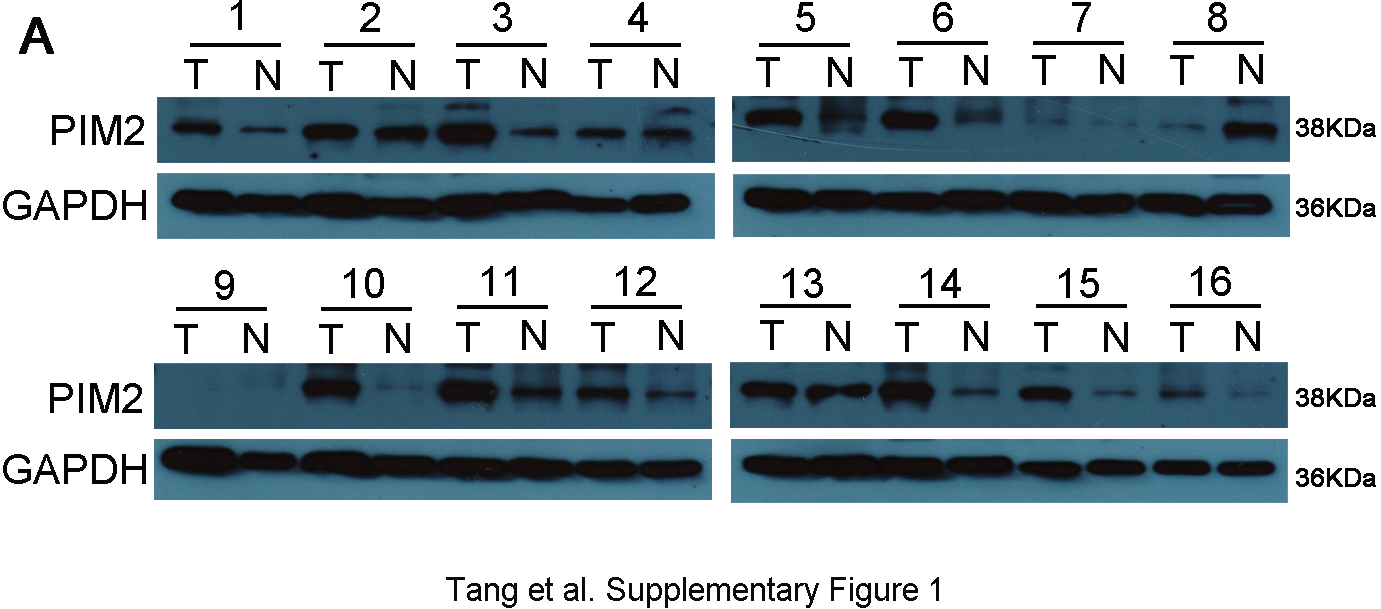

Supplement: Supplementary file 2 — Supplementary Figure 1. PIM2 expression in HCC clinical samples. [file 41419_2020_2700_MOESM2_ESM.tif]

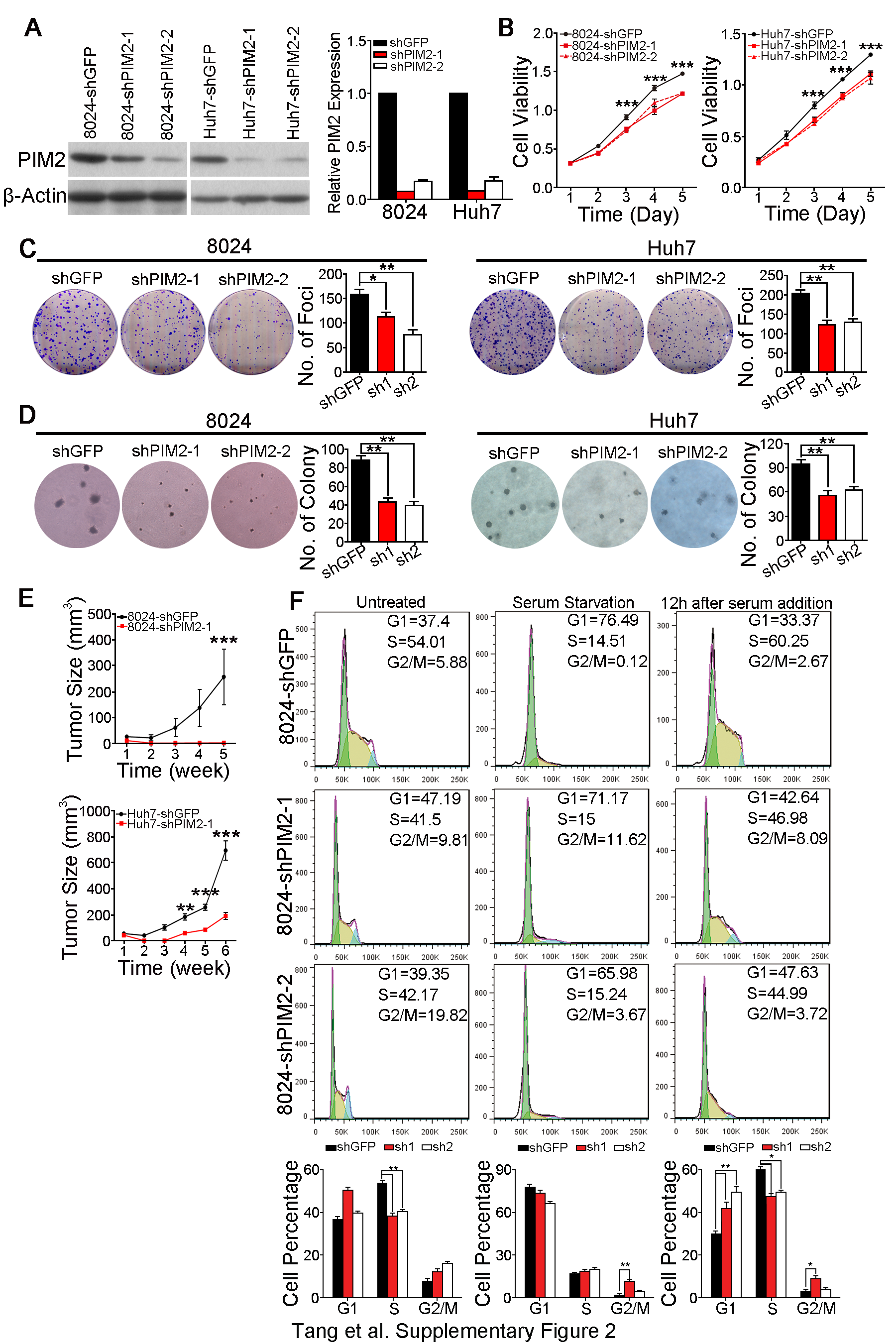

Supplement: Supplementary file 3 — Supplementary Figure 2. PIM2 knockdown attenuated HCC cells’ tumorigenic ability. [file 41419_2020_2700_MOESM3_ESM.tif]

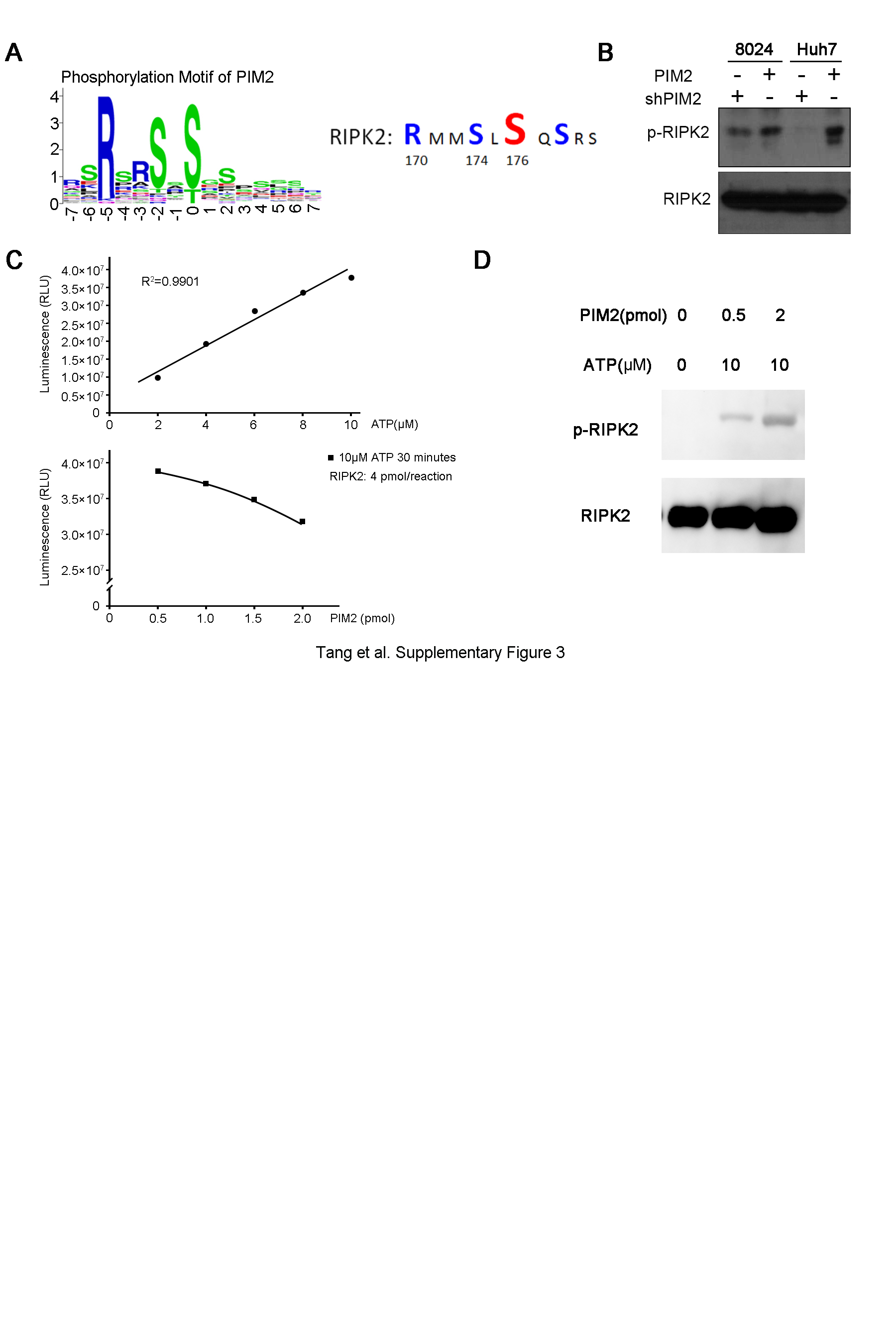

Supplement: Supplementary file 4 — Supplementary Figure 3. PIM2 can phosphorylate RIPK2 in vitro and vivo. [file 41419_2020_2700_MOESM4_ESM.tif]
